# Classical *versus* chemometrics tools for spectrophotometric determination of fluocinolone acetonide, ciprofloxacin HCl and ciprofloxacin impurity-A in their ternary mixture

**DOI:** 10.1186/s13065-023-00963-w

**Published:** 2023-06-07

**Authors:** Mahmoud A. Tantawy, Israa A. Wahba, Samah S. Saad, Nesrin K. Ramadan

**Affiliations:** 1 Analytical Chemistry Department, Faculty of Pharmacy, Cairo University, Kasr el Aini Street, Cairo, 11562 Egypt; 2 Chemistry Department, Faculty of Pharmacy, October 6 University, 6 October City, Giza, Egypt; 3 Pharmaceutical Analytical Chemistry Department, College of Pharmaceutical Sciences and Drug Manufacturing, Misr University for Science & Technology, 6th of October City, Giza, Egypt

**Keywords:** Fluocinolone acetonide, Ciprofloxacin HCl, Ciprofloxacin impurity-A, Spectrophotometry, Chemometrics

## Abstract

Green, simple, accurate and robust univariate and chemometrics assisted UV spectrophotometric approaches have been adopted and validated for concurrent quantification of fluocinolone acetonide (FLU), ciprofloxacin HCl (CIP) together with ciprofloxacin impurity-A (CIP imp-A) in their ternary mixture. Double-divisor ratio spectra derivative (DDRD) method has been used for determination of FLU. On the other hand, the first (D^1^) and second (D^2^) derivative approaches have been applied for the quantification of CIP and CIP imp-A, respectively. For the ratio difference (RD), derivative ratio (DR), and mean centering of ratio spectra (MC) methods, CIP and its impurity A have been simultaneously determined. The acquired calibration plots were linear over the concentration range of 0.6–20.0 μg/mL, 1.0–40.0 μg/mL and 1.0–40.0 μg/mL for fluocinolone acetonide, ciprofloxacin HCl, and ciprofloxacin impurity-A, respectively. The chemometrics methods namely; partial least squares (PLS) and artificial neural networks (ANN) were used for the concurrent determination of the three adopted components via using twenty-five mixtures as calibration set and fifteen mixtures as validation one. The investigated approaches were validated in accordance with International Council for Harmonisation (ICH) guidelines, and statistically compared with the official ones. The proposed methods were acceptably applied to the examination of FLU and CIP in their pure powders and pharmaceutical ear drops.

**Supplementary Information:**

The online version contains supplementary material available at 10.1186/s13065-023-00963-w.

## Introduction

Fluocinolone acetonide (FLU) is a corticosteroid medication, with a formula of 6-alpha, 9-alpha-difluoro-16-alpha, 17 alpha-acetonide [1]. It is used in for treatment of eczema, dermatitis and allergy [2]. The drug is official in British Pharmacopoeia (BP) [3] and United States Pharmacopoeia (USP) [4] where its determination was conducted through HPLC technique. FLU was determined via different analytical methods; spectrophotometric [5], HPLC [6–11], TLC [9] and capillary electrophoretic methods [12].

Ciprofloxacin HCl (CIP) is a fluoroquinolone antibiotic, chemically known as 1-cyclopropyl-6-fluoro-1,4-dihydro-4-oxo-7-(1-piperazinyl)-3-quinolinecarboxylic acid;hydrochloride [1]. It is used widely for the management of urinary tract’s infections, sinus’s infections, and pneumonia [13]. This cited drug is officially presented in BP [3] and USP [4]. CIP was assayed in BP by liquid chromatographic method with a TLC one as a limit test for its specified impurity A (CIP-imp A) [3]. Several analytical techniques were published for estimation of CIP including; spectrophotometry [14–17], HPLC [18–20], TLC [18, 21] and capillary electrophoresis [22–24]. Some chromatographic techniques have also been published for the determination of CIP in the existence of its impurities [25–27].

FLU and CIP combination is shown to be more efficacious in the treatment of otitis media than using a monotherapy strategy of each drug separately [28]. The two cited drugs are co-formulated together in otic solution, with a challengeable ratio of 1 FLU:12 CIP, for pediatric patients who are suffering from acute otitis media with tympanostomy tube otorrhea [29]. Considering the literature survey, it was found that FLU and CIP were simultaneously determined in their binary mixture by two spectrophotometric [28, 30] and one chromatographic methods [31]. No spectrophotometric technique has been reported yet for the assaying of this challengeable ratio dosage form along with CIP imp-A. Therefore, our efforts were directed to develop and validate simple, accurate, less expensive and less time consuming spectrophotometric methods for concurrent determination of the investigated compounds, FLU, CIP and CIP-imp A, in their ternary mixture. Classical univariate along with chemometrics assisted spectrophotometric methods were utilized and compared.

## Methods/experimental

### Instruments and software

Shimadzu1601 dual beam UV–Vis spectrophotometer (Kyoto, Japan) with 1-cm quartz cell. UV-Probe 2.21 software was used to manipulate absorption and derivative spectra. Absorption spectra were recorded in the range (200–400 nm) with the interval of 0.1 nm. Matlab 7.1 (2004) was used for analyzing the, data supplied with PLS tool box 2.1 and Neural Network tool box.

### Materials and reagents

#### Pure standards

FLU and CIP were obtained from Eva-Pharma and SEDICO (Egypt), respectively. Their respective potencies were estimated to be 100.20% ± 0.917 and 100.12% ± 0.758 [3]. CIP impurity was purchased from Sigma-Aldrich, Germany.

#### Pharmaceutical otic solution

Otovel®, 0.0625 mg FLU and 0.75 mg CIP per 0.25 mL, lot no. 20DE7, owned by Laboratories SALVAT (Barcelona, Spain), manufactured and distributed in USA by Arbor Pharmaceuticals (Atlanta, Georgia, USA).

#### Chemicals

Phosphoric acid (Adwic, Egypt), water of double distilled grade (Otsuka, Egypt), potassium hydroxide (Merck, Germany). Phosphate buffer solution (pH 3.6) was prepared by adding 750 μL phosphoric acid to 530 mL water, followed by pH adjustment with potassium hydroxide (10%) [1].

### Solutions

#### Stock solutions

FLU, CIP and CIP imp-A stock solutions, with respective concentrations of 40.0 μg/mL, 100.0 μg/mL100.0 μg/mL, were prepared in phosphate buffer (pH 3.6).

#### Laboratory prepared mixtures

A series of 10-mL measuring flasks were accurately filled with various aliquots from the three stock solutions. The prepared mixtures were diluted to the mark with buffer solution.

### Procedures

#### Construction of calibration curves for univariate methods

Different aliquots of stock solutions were precisely transferred to separated sets of 10-mL measuring flasks where volumes were adjusted to the mark using buffer solution. A 0.6–20.0 μg/mL concentration range for FLU, and 1.0–40.0 μg/mL concentration ranges for CIP and its impurity were obtained. Absorption spectra were then scanned against buffer solution in 200–400 nm range.

#### For determination of FLU

A Double-divisor ratio spectra derivative (DDRD) method has been used. The zero order absorption spectra of FLU (0.6–20.0 ﻿μg/mL), that were previously stored, were divided by a standard mixture of CIP and CIP imp-A (10.0 μg/mL, each in buffer solution) as a double divisor. First derivative of these ratio spectra was then computed (∆λ = 4 nm), and amplitudes at 251.4 nm were recorded for determining FLU.

#### For simultaneous determination of CIP and CIP imp-A

The absorption spectra of CIP and its impurity A (1.0–40.0 μg/mL) were recorded within 200 to 400.0 nm range. Different spectrophotometric methods were the applied to resolve their overlapped spectra in the range of 308.0–370.0 nm where no influence from FLU spectrum observed. These methods encompasses: first derivative (D^1^), second derivative (D^2^), ratio difference (RD), derivative ratio (DR), and mean centering of ratio spectra (MC).

##### ***First (D***^***1***^***) and second (D***^***2***^***) derivative methods***

D^1^ spectra of CIP (1.0–40.0 ﻿μg/mL) were recorded using 10 as a scaling factor and Δλ of 4. The peak amplitudes of the resulting spectra were observed at 320.7 nm. On the other hand, D^2^ spectra of CIP imp-A (1.0–40.0 μg/mL) were obtained considering a Δλ of 4 nm and a scaling factor of 100. The acquired peak amplitudes were measured at 335.1 nm.

##### Ratio difference (RD) method

For CIP, ratio spectra were obtained through dividing the scanned CIP (1.0–40.0 μg/mL) spectra by the absorption spectrum of CIP imp-A (1.0 μg/mL) as a divisor. The differences between ratio spectra amplitudes were calculated at 313.9 nm and 335.8 nm. For CIP imp-A, ratio spectra were acquired via dividing the scanned spectra of CIP imp-A (1.0–40.0 μg/mL) by CIP absorption spectrum (7.0 μg/mL). CIP imp-A was determined using the differences in ratio spectrum amplitudes between 328.3 nm and 307.8 nm.

##### Derivative ratio (DR) method

The derivative ratio spectra of CIP were obtained by first derivatizing the previously stored ratio spectra of CIP with respect to scaling factor = 10 and ∆λ = 4 nm. 331.9 to 340.5 nm (peak to peak) amplitudes were then recorded. Whereas, the formerly saved ratio spectra of CIP imp-A were first differentiated using the previously mentioned parameters. Peak to peak amplitudes of its DR spectra were estimated at 332.1 to 339.2 nm.

##### Mean centering of ratio spectra (MC) method

The previously obtained ratio spectra of CIP and CIP imp-A were separately mean centered via Matlab^®^[32] software. The mean-centered values of CIP and CIP imp-A were measured at 345.2 and 335.6 nm, respectively.

#### Chemometrics assisted partial least squares (PLS) and artificial neural networks (ANN) methods

Multilevel multifactor design, developed by Brereton, was followed [33]. The calibration set's absorption spectra for twenty-five different laboratory prepared mixtures, for the three components in different ratios, were recorded in 210.0–270.0 nm range at 0.2 nm interval. The obtained 301 experimental points were moved to Matlab® for further analysis, and calibration models construction. Prior to calibration, all the numbers were mean centered. Fifteen validation mixture were prepared separately where the optimized PLS and ANN calibration models were used to determine the concentrations of each cited component.

#### Application to pharmaceutical ear drops and application of standard addition technique

Five vails of Otovel^®^ were emptied, and 0.6 mL of the otic solution was precisely put into 10-mL measuring flask. The volume was completed with buffer solution to attain concentration of 0.015 mg/mL for FLU and 0.18 mg/mL for CIP. Aliquots of 5.0 mL from the prepared solution were accurately introduced to two 100-mL measuring flasks, and diluted to the mark with buffer solution to make dosage form solution with claimed concentrations of 0.75 µg/mL and 9.0 µg/mL for FLU and CIP, respectively. The two drugs concentrations were determined using their corresponding regression equations following the execution of the general procedures previously outlined for each approach.

## Results and discussion

**Fig. 1:**
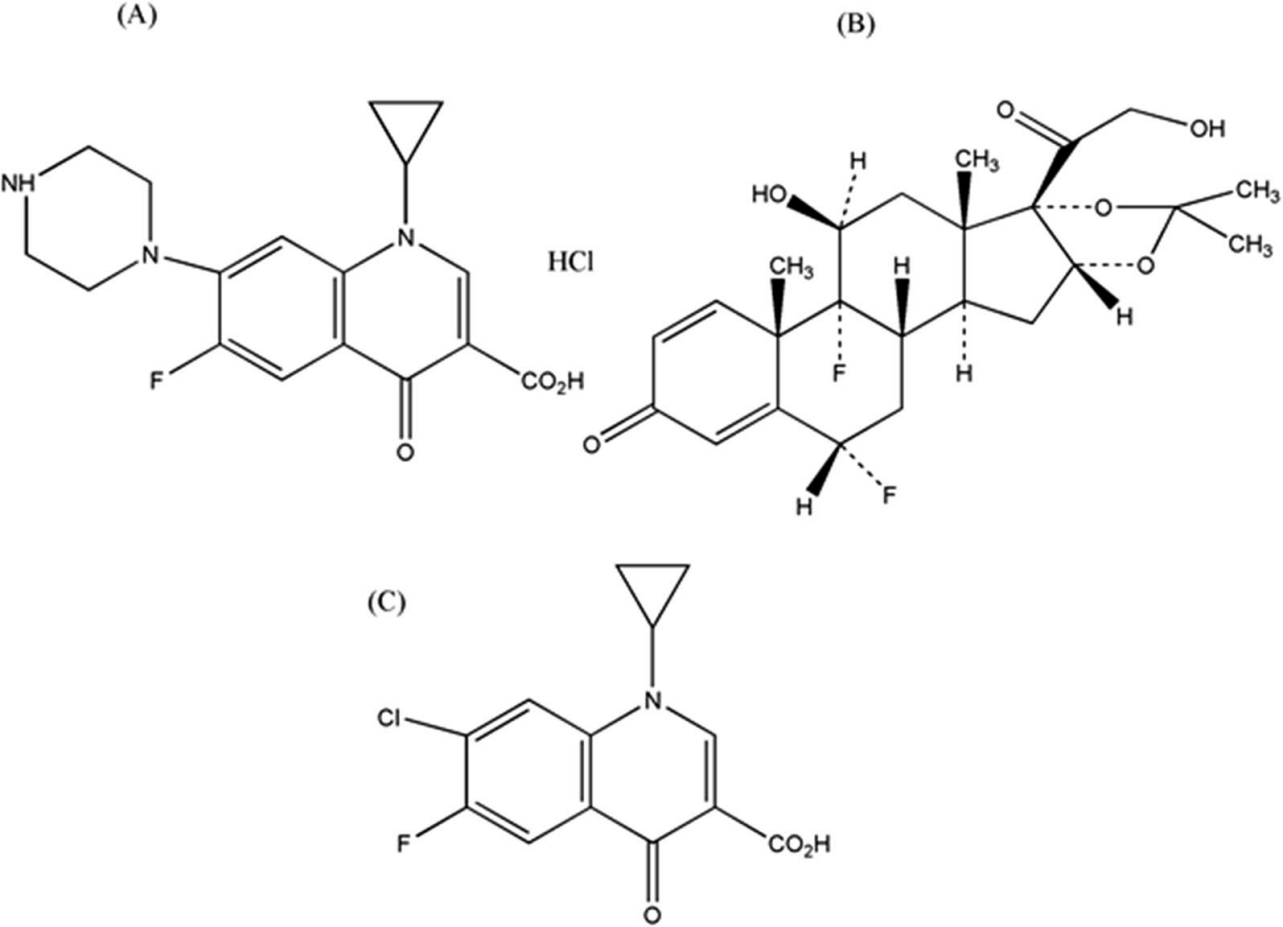
Chemical structures of **A** ciprofloxacin HCl, **B** fluocinolone Acetonide and **C** ciprofloxacin impurity A

**Fig. 2:**
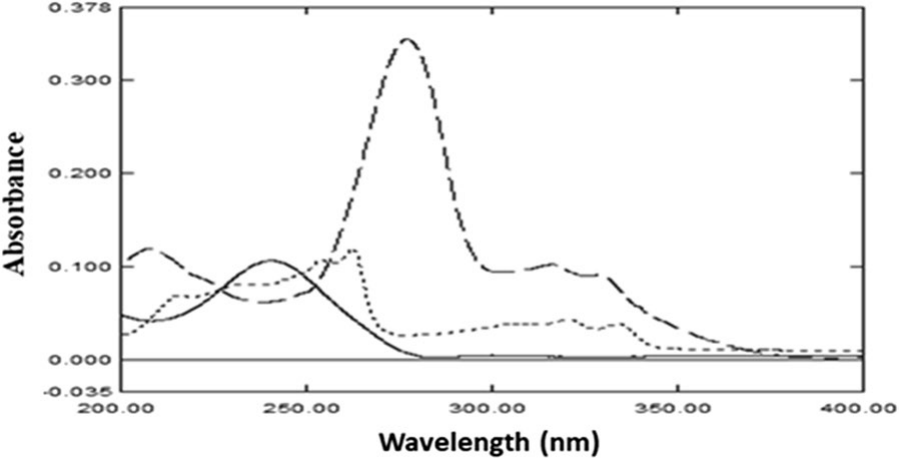
Zero order of 4.0 μg/mL ciprofloxacin HCl (---), 2.4 μg/mL fluocinolone acetonide (—) and 1.0 μg/mL ciprofloxacin impurity-A (……)

The goal of this work was to create precise, accurate, easy-to-use, and robust spectrophotometric methods for determining FLU, CIP, and CIP imp-A concurrently in pharmaceutical formulation and pure powders. The greenness of the methods was prioritized through avoiding organic solvents. Buffer of pH 3.6 was chosen to simulate the studied otic solution pH. The three components’ chemical structures are presented in Fig. 1. By observing the zero order absorption spectra of the three investigated components, spectral overlap was noticed which hindered their direct determination, Fig. 2. A selective and sensitive determination of FLU, CIP and CIP imp-A could be achieved by applying the suggested spectrophotometric methods without preliminary separation step.

### Classical univariate methods

#### Determination of FLU by DDRD method

**Fig. 3:**
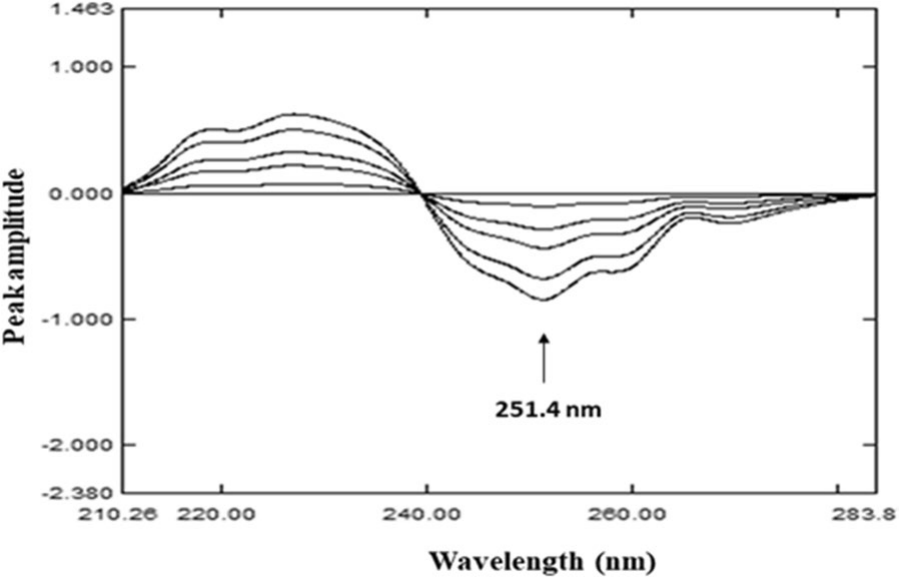
Double divisor ratio spectra derivative (DDRD) of 0.6–20.0 μg/mL FLU using 10.0 ﻿μg/mL CIP + 10.0 ﻿μg/mL CIP imp-A as double divisor

This method is based on determination of one drug in its ternary mixture through derivatizing the ratio spectra acquired via dividing the absorption spectrum of that drug by sum of the other two components spectra [34]. In this work, DDRD method was developed for determining of FLU in presence of CIP and CIP imp-A without prior separation step. The absorption spectra of FLU were divided by the sum of CIP and CIP imp-A spectra, 10.0 μg/mL each, as a double divisor. Those ratio spectra were then differentiated, and FLU was quantified through measuring peak amplitudes at 251.4 nm Fig. 3. Different concentrations of CIP and CIP imp-A (1.0, 3.0, 5.0 and 10.0 μg/mL) were tasted in order to optimize the suggested DDRD method. It is worth noting that using a mixture of CIP and CIP imp-A of 10.0 μg/mL each as a divisor led to the lowest possible noise level and high selectivity. A calibration curve relating peak amplitude to the corresponding FLU concentrations in the range of 0.6–20.0 µg/mL was plotted. The results of the regression equation calculation are shown in Table 1.

**Table 1:** Assay parameters and method validation for the determination of ciprofloxacin HCl, fluocinolone acetonide and ciprofloxacin impurity A by the proposed spectrophotometric methods according to ICH guidelines

Parameter	CIP				FLU	CIP imp-A			
	D^1^	RD	DR	MC	DDRD	D^2^	RD	DR	MC
Range	(1–40) μg/mL				(0.6–20) μg/mL	(1–40) μg/mL			
Slope	0.0054	0.2424	1.2969	0.5433	0.0423	0.0161	0.005	0.0727	0.0197
Intercept	0.0021	0.0764	− 0.1976	0.0433	0.0029	0.0546	0.0132	0.1897	− 0.0078
SE slope	3.6949 × 10^–5^	1.4273 × 10^–3^	8.0659 × 10^–3^	2.3690 × 10^–3^	3.0186 × 10^–4^	1.5795 × 10^–4^	9.8110 × 10^–5^	9.0012 × 10^–4^	1.0932 × 10^–3^
SE intercept	9.0549 × 10^–4^	0.0350	0.1977	0.0581	3.2484 × 10^–3^	3.8317 × 10^–3^	2.5945 × 10^–3^	0.0238	0.0287
Correlation coefficient (r)	0.9998	0.9999	0.9999	0.9999	0.9998	0.9996	0.9995	0.9996	0.9996
LOD (μg/mL)	0.281	0.210	0.202	0.196	0.193	0.329	0.325	0.312	0.287
LOQ (μg/mL)	0.852	0.636	0.612	0.594	0.585	0.997	0.985	0.945	0.870
Accuracy^a^ ± SD	100.19 ± 0.871	101.02 ± 0.917	100.94 ± 1.564	100.66 ± 1.146	99.54 ± 1.293	100.09 ± 1.353	100.31 ± 0.792	99.32 ± 0.925	99.25 ± 1.081
Robustness ^b^ (RSD%)									
Wavelength	1.060	1.226	1.851	1.141	1.825	1.691	1.606	0.820	1.220
Buffer pH	0.963	1.032	1.056	0.874	1.281	1.630	1.613	0.435	1.165
KOH %	0.736	0.837	1.013	0.523	0.648	1.539	0.847	0.385	0.748
Repeatability ^c^ (RSD%)	1.214	1.441	1.693	1.021	1.506	1.548	1.281	1.225	1.274
Intermediate ^d^ precision (RSD%)	1.391	1.739	1.950	1.556	1.787	1.941	1.715	1.728	1.866

^a^Average of five determinations

^b^Robustness (n = 9), average of three concentrations of FLU (1, 4, 5.5.0 µg/mL), CIP (17.0, 20.0, 30.0 µg/mL) and CIP imp- A (7.0, 16.0, 27.0 µg/mL) repeated three times

^c^Repeatability (n = 9), average of three concentrations of FLU (1.1, 3.5, 5.0 µg/mL), CIP (5.0, 12.0, 33.0 µg/mL) and CIP imp- A (3.0, 12.0, 20.0 µg/mL) repeated three times within the day (intra-daily)

^d^The inter-daily precision (n = 9), average of three concentrations of FLU (1.1, 3.5, 5.0 µg/mL), CIP (5.0, 12.0, 33.0 µg/mL) and CIP imp- A (3.0, 12.0, 20.0 µg/mL) repeated three times on three successive days

#### Simultaneous determination of CIP and CIP imp-A

As shown in Fig. 2, the sever overlap between absorption spectra of CIP and CIP imp-A, in the range of 300–400 nm, hindered their direct determination. Therefore, different spectrophotometric methods were established for concurrent quantification of CIP and its impurity A in that specified range. The proposed methods were based on D^1^, D^2^, RD, DR and MC techniques.

##### ***D***^***1***^*** and D***^***2***^*** methods***

For CIP determination, the stored spectra were firstly derivatized with a scale factor of 10 and ∆λ of 4 nm. The obtained spectra demonstrated that CIP was detectable at 320.7 nm without interference from CIP imp-A, Additional file 1: Figure S1. Calibration curve was developed through relating peak amplitudes to the corresponding CIP concentrations. The parameters of linear regression were computed, as stated in Table 1. On the other hand, D^2^ method was utilized for determining CIP imp-A. The stored spectra were secondly derivatized using Δλ = 4 nm and scale factor of 100. Peak amplitudes at 335.1 nm were plotted against the corresponding concentrations of CIP imp-A, Additional file 1: Figure S2. Regression parameters were estimated, Table 1. Those methods are characterized by good selectivity, sensitivity and simplicity whereas critical step is the choice of a wavelength of no contribution for interfering drug.

##### RD method

This method depends on division of the absorption spectrum of the target analyte over the interfering component spectrum. The corresponding concentration of the desired analyte will be directly proportional to the amplitude difference (ΔP) between two different wavelengths [35]. Different concentrations of CIP (1.0, 5.0, and 7.0 μg/mL) and CIP imp-A (1.0, 3.0, and 4.0 μg/mL) were tested as divisors in this binary mixture. The optimal divisor for the CIP quantification was 1.0 μg/mL of CIP imp-A., Additional file 1: Figure S3. For CIP imp-A, a divisor of 7.0 μg/mL CIP was utilized, Additional file 1: Figure S4. Linearity was obtained by measuring ΔP between 313.9 and 335.8 nm for CIP determination. On the other hand, good CIP imp-A linearity was achieved via relating ΔP values between 307.8 and 328.3 nm to its corresponding concentration. As shown in Table 1, linear regression equations were figured. This adopted method is simple, rapid, accurate and more robust concerning small wavelength variations.

##### DR method

**Fig. 4:**
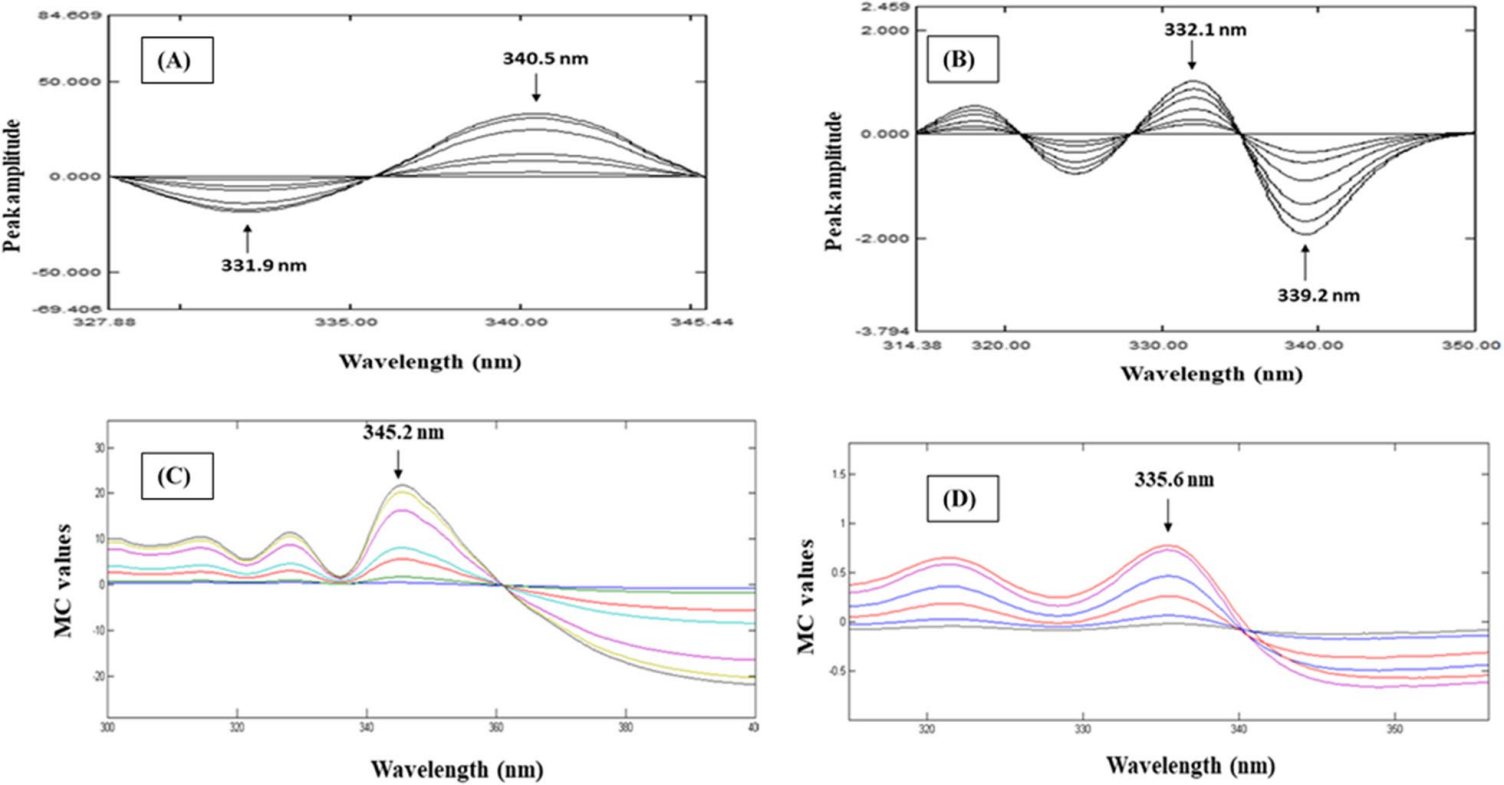
Derivative ratio spectra of; **A** 1.0–40.0 μg/mL CIP using 1.0 μg/mL CIP imp-A as a divisor and **B** 1.0–40.0 μg/mL CIP imp-A using 7.0 μg/mL CIP as a divisor. Mean centering ratio spectra of; **C** 1.0–40.0 μg/mL CIP and **B** 1.0–40.0 μg/mL CIP imp-A

This method is depending on derivatizing the previously stored ratio spectra. This approach revokes the whole spectrum of the interfering drug [36, 37]. Different variables comprise divisor concentration, wavelength increment (∆λ) and smoothing factor should be carefully studied for optimization of this method to minimize reading error in the signal. Derivatization of the formerly stored ratio spectra for both of CIP and CIP imp-A was performed using a scaling factor of 10 and ∆λ = 4. Good linearity was obtained at peak_331.9 nm_ to peak_340.5 nm_ values for CIP, Fig. 4A. For CIP imp-A, values at peak_332.1 nm_ to peak_339.2 nm_ were measured, Fig. 4B. For CIP and CIP imp-A, the linear regression equations were constructed, as shown in Table 1. The main advantage of DR is that the entire interfering analyte spectrum is canceled out by derivatization whereas its drawback is numerous manipulating steps of divisor selection, division and derivatization.

##### MC method

MC method stands on mean centering, as a mathematical operation, of ratio spectra. This mathematical operation excludes the derivative phase and improves signal-to-noise ratio [38–40]. In this work, MATLAB^®^ 7.0.1 [32] was used to perform such calculations for CIP and CIP imp-A determinations. Good correlation was obtained through plotting the mean centered values of CIP and CIP imp-A at 345.2 and 335.6 nm, respectively, versus their corresponding concentrations, Fig. 4C, D. The estimated values for the linear regression equations are shown in Table 1. This method has advantage of automated nature and time saving whereas its main obstacle is the need of MATLAB^®^ software to manipulate the ratio spectra.

### Chemometrics assisted methods

Chemometrics tools are usually applied for multivariate spectral analysis of pharmaceutical mixtures comprising two or more drugs with severely overlapping spectra where no necessity for separation steps before determination [33, 41–43]. Two multivariate chemometrics methods, namely; PLS and ANN, were conducted in this work for synchronous quantification of FLU, CIP and CIP imp-A. In these techniques, calibration was accomplished by using the absorbance and concentration data matrices to predict the unknown concentrations of the three cited components in their ternary mixtures. UV spectra of twenty five mixtures and fifteen mixtures were scanned and stored over 210.0–270.0 nm range to calibrate and validate the proposed models. Wavelengths larger than 270.0 nm were omitted since CIP and CIP imp-A exhibit the same absorbance characteristics in this range and, therefore, are less useful. Wavelengths lower than 210.0 nm were excluded due to strong noise influence.

#### PLS

**Fig. 5:**
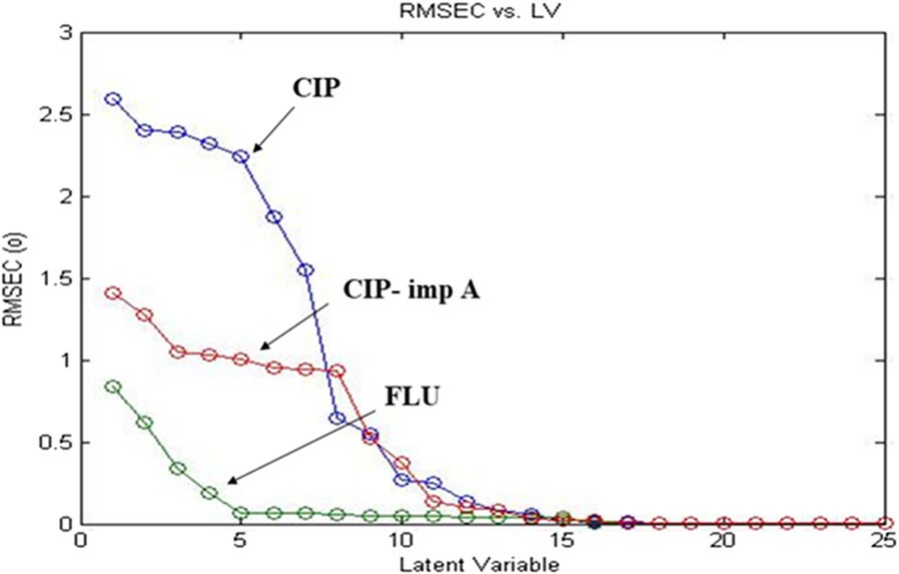
Root mean square error of calibration versus the number of latent variables used to construct the PLS model

PLS is the most widely used chemometrics method for constructing multivariate calibration sets [44]. In order to build PLS model, cross-validation step, of leaving one sample out each time, was applied. Optimal number of latent variables selection was achieved a according to Haaland and Thomas criteria [45] where the least significant prediction error was characterized by the application of five latent variables, Fig. 5.

#### ANN

Three layers are present for an ANN: (a) Input, (b) hidden, and (c) output layers, with transfer functions [46]. 301 neurons were used in the input layer, which correspond to the number of spectral data points used. Three neurons were employed in the output layer, one for each component that needed to be determined for each sample. On a trial-and-error basis, the hidden layer’s neuron number should be adjusted. RMSEC values were significantly decreased from 2 to 4 hidden neurons while the decline became negligible upon further incrementing in the hidden neurons’ numbers. Four hidden neurons with purelin-purelin transfer function was the optimal condition. In addition, 50 epochs and a learning rate of (0.1) were set up.

### Methods validation

The suggested methods were validated as per ICH recommendations [47].

#### Classical univariate methods

##### Linearity and range

The linearity of the investigated methods was assessed via examining 0.6–20.0 μg/mL for FLU, and 1.0–40.0 μg/mL for CIP and its impurity A. Analyses of those three components were performed as per the conditions formerly provided under each method, Table 1.

##### Limits of detection (LODs) and limits of quantitation (LOQs) calculation

The obtained calibration plots were utilized to deduce the standard deviation of residuals’ values for each method. After that, the calculation of LOD and LOQ was conducted for each component, using their respective equations, Table 1.

##### Accuracy

For accuracy assessment, the adopted methods were used to analyse five concentrations of pure FLU, CIP, and CIP imp-A. The mean percentage recoveries for each drug, as shown in Table 1, indicated that the proposed procedures were accurate.

##### Precision

Repeatability, three concentrations of pure FLU (1.1, 3.5, 5.0 µg/mL), CIP (5.0, 12.0, 33.0 µg/mL) and CIP imp- A (3.0, 12.0, 20.0 µg/mL) were assessed 3 times intraday. Relative standard deviations (RSD%) at these concentration levels were computed and values show great repeatability and minimal deviation, Table 1.

Intermediate precision, it was expressed through analyzing the three elected concentrations interdaily. According to the calculations shown in Table 1, good precision was achieved.

##### Robustness

By measuring the peak amplitude for each accepted method at the given wavelength ± 0.2 nm using various buffers with pH values of 3.4, 3.6, 3.8, and 4.0, three concentrations of each of FLU, CIP, and CIP imp-A were determined. All methods were confirmed to be robust and the RSD% was found to be below 2.0%, as shown in Table 1. The table also assures methods’ robustness towards changing the concentration of potassium hydroxide used for buffer preparation by ± 1%.

##### Specificity

FLU, CIP, and CIP imp-A laboratory mixtures were formed in a variety of ratios through their specified ranges, and quantified using the suggested methods. The three aforementioned components were determined independently of one another where the results represented in Additional file 1: Table S1 ensure the specificity of adopted methods.

#### Chemometrics assisted methods

Several diagnostic approaches were used to examine the established PLS and ANN models' ability for prediction. For each component, the average recoveries and the RSD% were computed, as shown in Additional file 1: Table S2. Moreover, regression parameters of the validation sets, and root mean square error of prediction (RMSEP) values were estimated, Table 2.

**Table 2:** Regression parameters of the validation sets calculated for each proposed model

Component	Model	Slope	Intercept	LOD (µg/ mL)	LOQ (µg /mL)	R	RMSEP
CIP	PLS	0.9983	0.0249	0.192	0.582	0.9997	0.231717
	ANN	1.0000	-0.0001	0.007	0.021	0.9999	0.002036
FLU	PLS	1.0160	-0.0330	0.081	0.245	0.9998	0.027706
	ANN	1.0026	-0.0028	0.096	0.290	0.9997	0.028292
CIP imp-A	PLS	0.9822	0.0680	0.202	0.606	0.9995	0.062250
	ANN	1.0065	-0.0073	0.002	0.006	0.9996	0.056662

### ***Application to Otovel***^***®***^*** ear drops***

The investigated spectrophotometric and chemometrics approaches were successfully utilized for quantification of FLU and CIP in its pharmaceutical formulations (Otovel^®^). Moreover, validity and suitability of those models were assessed via applying standard addition technique, Table 3.

**Table 3:** Determination of CIP and FLU in its otic vial dosage form and application of standard addition technique using the suggested methods

Otovel® otic vials 0.75 mg CIP/ 0.0625 mg FLU in each 0.25 mL lot no. 20DE7		CIP						Otovel® otic vials 0.75 mg CIP/ 0.0625 mg FLU in each 0.25 mL lot no. 20DE7		FLU		
		D^1^	RD	DR	MC	PLS	ANN			DDRD	PLS	ANN
Mean^a^		99.61	100.97	99.64	100.08	100.09	99.43	Mean^a^		100.26	98.65	100.41
RSD%		1.637	1.388	1.659	1.309	1.027	1.159	RSD%		1.205	1.281	1.042
Standard addition technique												
CIP								FLU				
Taken (μg/mL)	Added (μg/mL)	Recovery %						Taken (μg/mL)	Added (μg/mL)	Recovery %		
9.4	5.0	99.26	99.32	99.98	101.91	98.82	98.72	0.78	0.4	99.92	99.28	101.21
	10.0	101.11	101.56	101.38	99.49	101.32	100.52		0.8	100.20	100.12	100.94
	20.0	101.48	98.02	99.87	100.35	101.01	99.02		1.6	101.81	98.04	98.85
Mean		100.62	99.63	100.41	100.58	100.38	99.42	Mean		100.64	99.14	100.33
RSD%		1.183	1.799	0.840	1.218	1.358	0.970	RSD%		1.016	1.055	1.287

^a^Average recoveries of 5 determinations of tablet dosage form

### Statistical analysis

The suggested analytical methods were compared to the official ones [3]. Both student’s t-test and F-test were conducted, and calculated values were less than the theoretical ones. As a result, there is no pronounced difference between the compared methods, Additional file 1: Table S3.

### *Evaluation of methods greenness *via* analytical GREEnness Metric (AGREE)*

The software for this metric is freely provided by Pena-Pereira et al. [48]. The method’s inputs yield a chart with twelve sectors, each ranging in color from deep green to deep red. The overall score ranging from 0.00 (not green) to 1.00 (greenest) is presented in the middle of that chart. This score is calculated based on 12 Green Analytical Chemistry (GAC) principles [49–51]. To prove the superiority of our method compared to the two published spectrophotometric approaches [28, 30] for the simultaneous CIP and FLU assay, the linearity ranges, types of analyzed samples, solvents used, and AGREE scores were compared, Table 4. As shown in that table, our method’s sustainability is assured with a 0.88 score beside the widest linearity range obtained as well as the successful determination of CIP imp-A.

**Table 4:** Greenness assessment by AGREE tool and methods comparison

Method	Linearity range^a^ (μg/mL)		Analyzed samples	Solvent used	AGREE assessment
	CIP	FLU			
Reference [28]	1–15		CIP and FLU	Methanol	
Reference [30]	﻿3–15				
This work	1–40	0.6–20	CIP, FLU and CIP imp-A	Phosphate buffer, pH 3.6	

^a^The widest range was chosen for each work

## Conclusion

The present work provides spectrophotometric approaches for concurrent determination of fluocinolone acetonide and ciprofloxacin HCl in their formulations. Also, this work ensures the ability of the investigated methods for detection and determination of ciprofloxacin impurity A in a pharmaceutical dosage form containing ciprofloxacin as an active ingredient. In spite the successfulness of the classical univariate approaches to determine the three studied drugs, they are time consuming and need many mathematical procedures. On the other hand, the two proposed multivariate models, PLS and ANN, require less time and steps to simultaneously determine those studied components. Moreover, PLS and ANN models successfully detect lower concentrations contrary to the univariate ones. The high ANN model’s predicting ability is manifested in detecting ciprofloxacin impurity A up to 0.002 μg/mL which exceeds its pharmacopoeial limit of 0.2%. Those analytical methods could be used for impurity profiling of the two cited drugs in future studies. All the adopted methods follow the green principles of using a non-hazardous phosphate buffer as a solvent. They could be also applied for the routine analysis of fluocinolone acetonide and ciprofloxacin HCl in their combined Otovel^®^ ear drops of a challengeable ratio.

## Supplementary Information

**Additional file1: Figure S1.** First order derivative spectra of (1.0–40.0 μg/mL) CIP. **Figure S2.** Second order derivative spectra of (1.0- 40.0 μg/mL) CIP imp-A. **Figure S3.** Ratio spectra of (1.0–40.0 μg/mL) CIP using (1.0 μg/mL) CIP imp-A as a divisor. **Figure S4.** Ratio spectra of (1.0–40.0 μg/mL) CIP imp-A using (7.0 μg/mL) CIP as a divisor. **Table S1**. Determination of ciprofloxacin HCl, fluocinolone acetonide and ciprofloxacin impurity A in laboratory prepared mixtures by the proposed spectrophotometric methods. **Table S2.** Prediction recoveries of validation set samples. **Table S3.** Statistical comparison for the results obtained by the suggested methods and the reported method for the analysis of CIP and FLU.

## Data Availability

The datasets used and/or analysed during the current study are available from the corresponding author on reasonable request.

## Abbreviations

ANN: Artificial neural networks
BP: British pharmacopoeia
CIP: Ciprofloxacin hydrochloride
CIP imp-A: Ciprofloxacin impurity A
D^1^: First derivative
D^2^: Second derivative
DDRD: Double-divisor ratio spectra derivative
DR: Derivative ratio
FLU: Fluocinolone acetonide
HPLC: High performance liquid chromatography
ICH: International Council for Harmonisation
MC: Mean centering of ratio spectra
PLS: Partial least squares
RD: Ratio difference
TLC: Thin layer chromatography
USP: United States pharmacopoeia
