# Supplementary material for: Classical versus chemometrics tools for spectrophotometric determination of fluocinolone acetonide, ciprofloxacin HCl and ciprofloxacin impurity-A in their ternary mixture
Source: BMC Chem. 2023 Jun 7;17(1):49. doi: 10.1186/s13065-023-00963-w (PMC10249224; doi:10.1186/s13065-023-00963-w)
Supplement: Supplementary file 1 — Additional file1: Figure S1. First order derivative spectra of (1.0–40.0 μg/mL) CIP. Figure S2. Second order derivative spectra of (1.0- 40.0 μg/mL) CIP imp-A. Figure S3. Ratio spectra of (1.0–40.0 μg/mL) CIP using (1.0 μg/mL) CIP imp-A as a divisor. Figure S4. Ratio spectra of (1.0–40.0 μg/mL) CIP imp-A using (7.0 μg/mL) CIP as a divisor. Table S1. Determination of ciprofloxacin HCl, fluocinolone acetonide and ciprofloxacin impurity A in laboratory prepared mixtures by the proposed spectrophotometric methods. Table S2. Prediction recoveries of validation set samples. Table S3. Statistical comparison for the results obtained by the suggested methods and the reported method for the analysis of CIP and FLU. [file 13065_2023_963_MOESM1_ESM.docx]

Additional file

For

**Classical *versus* chemometrics tools for spectrophotometric determination of fluocinolone acetonide, ciprofloxacin HCl and ciprofloxacin impurity-A in their ternary mixture**

Mahmoud A. Tantawy, Israa A. Wahba, Samah S. Saad, Nesrin K. Ramadan


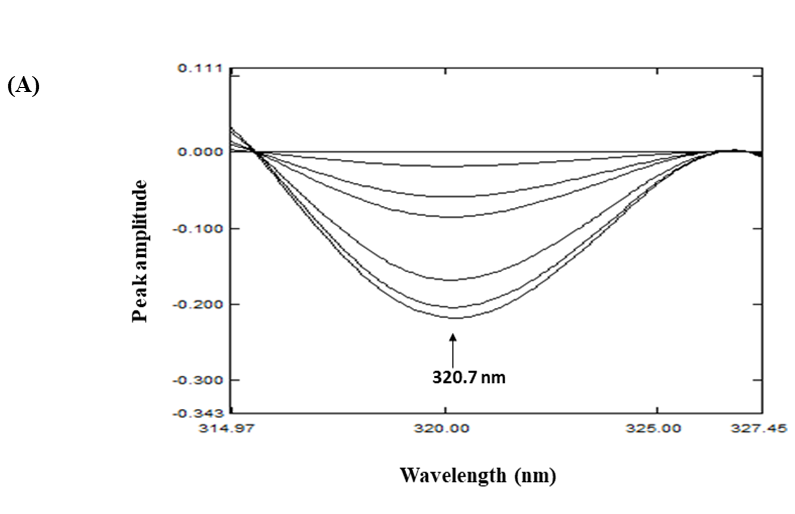


**Figure S1.** First order derivative spectra of (1.0-40.0 μg/mL) CIP.


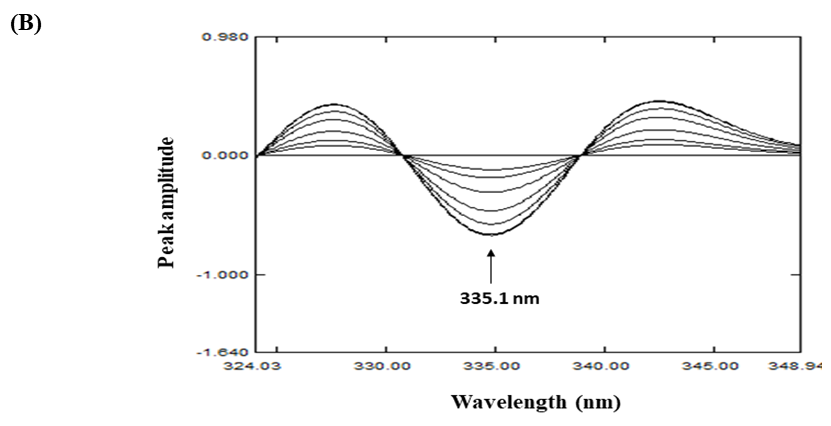


**Figure S2****.** Second order derivative spectra of (1.0- 40.0 μg/mL) CIP imp-A.


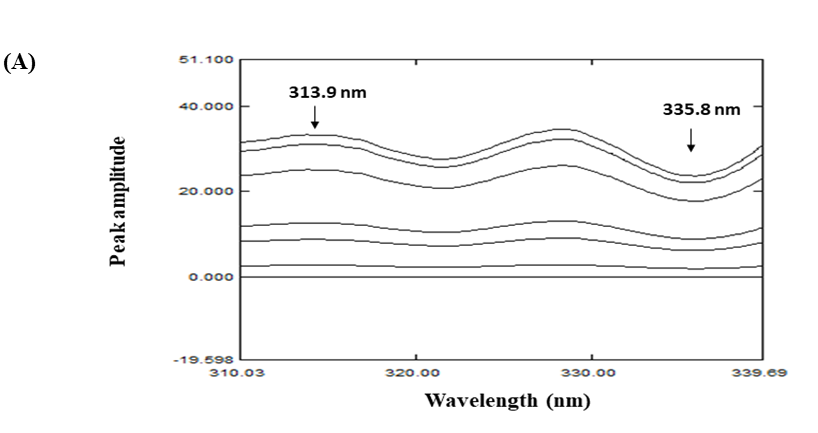


**Figure S3.** Ratio spectra of (1.0-40.0 μg/mL) CIP using (1.0 μg/mL) CIP imp-A as a divisor.


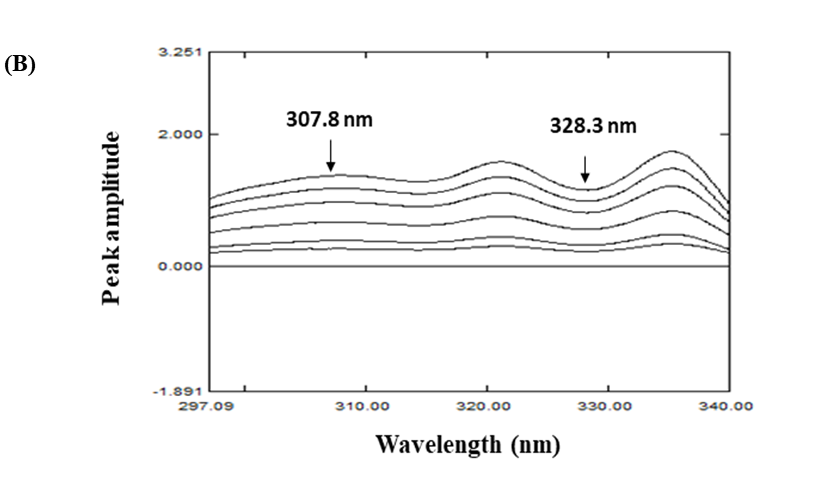


**Figure S4.** Ratio spectra of (1.0-40.0 μg/mL) CIP imp-A using (7.0 μg/mL) CIP as a divisor.

**Table S1**. Determination of ciprofloxacin HCl, fluocinolone acetonide and ciprofloxacin impurity A in laboratory prepared mixtures by the proposed spectrophotometric methods.

| **No. of mixture** | **Claimed concentration taken (µg/mL)** | | | **CIP** | | | | **FLU** | **CIP imp-A** | | | | |
| --- | --- | --- | --- | --- | --- | --- | --- | --- | --- | --- | --- | --- | --- |
|  | **CIP** | **FLU** | **CIP imp-A** | **D^1^** | **RD** | **DR** | **MC** | **DDRD** | **D^2^** | **RD** | **DR** | **MC** |  |
| 1 | 37.5 | 3.1 | 15 | 101.68 | 100.75 | 99.64 | 101.0 | 99.98 | 100.79 | 101.07 | 102.0 | 99.73 |  |
| 2 | 20.0 | 3.1 | 2.0 | 100.83 | 100.38 | 99.72 | 98.63 | 101.50 | 98.14 | 98.36 | 98.26 | 100.51 |  |
| 3 | 10.0 | 3.1 | 10.0 | 99.81 | 98.87 | 101.36 | 101.12 | 99.98 | 99.63 | 101.60 | 101.97 | 101.73 |  |
| 4 | 18.8 | 1.6 | 5.0 | 99.39 | 101.90 | 98.24 | 98.27 | 99.14 | 98.63 | 99.20 | 99.94 | 102.34 |  |
| 5 | 10.0 | 1.6 | 5.0 | 99.81 | 102.17 | 101.65 | 99.74 | 100.62 | 98.63 | 99.60 | 100.77 | 98.54 |  |
| 6 | 5.0 | 1.6 | 2.0 | 99.63 | 99.30 | 98.78 | 90.33^a^ | 99.14 | 101.86 | 99.01 | 100.83 | 101.02 |  |
| 7 | 12.5 | 1.0 | 5.0 | 102.07 | 101.37 | 98.92 | 99.84 | 99.53 | 100.21 | 98.67 | 101.93 | 101.32 |  |
| 8 | 9.4 | 0.78 | 10.0 | 100.28 | 101.80 | 99.37 | 99.79 | 100.32 | 101.49 | 97.80 | 99.08 | 98.88 |  |
| **Mean** | | | | 100.44 | 100.82 | 99.71 | 99.77 | 100.03 | 99.92 | 99.41 | 100.60 | 100.51 |  |
| **S.D.** | | | | 0.995 | 1.227 | 1.212 | 1.072 | 0.796 | 1.397 | 1.311 | 1.409 | 1.354 |  |
| **RSD%** | | | | 0.991 | 1.217 | 1.215 | 1.074 | 0.796 | 1.398 | 1.319 | 1.401 | 1.347 |  |

^a^ Rejected value [1]: the calculated Q = 0.736 for the value 90.33 which is more than the tabulated one (0.526) at confidence level = 95%

**Table S2.** Prediction recoveries of validation set samples.

| **Mix no** | **CIP** | | | **FLU** | | | **CIP IMP-A** | | |
| --- | --- | --- | --- | --- | --- | --- | --- | --- | --- |
|  | **Actual conc.**  **(µg/ mL)** | **PLS** | **ANN** | **Actual conc.**  **(µg/ mL)** | **PLS** | **ANN** | **Actual conc.**  **(µg/ mL)** | **PLS** | **ANN** |
| 1 | 11.0 | 100.90 | 99.99 | 1.8 | 98.5 | 98.01 | 3.0 | 102.86 | 101.87 |
| 2 | 11.0 | 102.84 | 100.03 | 0.6 | 95.96 | 102.25 | 2.0 | 101.35 | 98.28 |
| 3 | 1.0 | 103.50 | 99.77 | 1.2 | 99.65 | 98.28 | 1.0 | 98.42 | 101.31 |
| 4 | 6.0 | 102.29 | 99.99 | 0.6 | 97.86 | 102.26 | 5.0 | 100.72 | 98.01 |
| 5 | 1.0 | 97.62 | 100.26 | 3.0 | 99.82 | 100.5 | 5.0 | 98.73 | 98.06 |
| 6 | 21.0 | 100.75 | 100.01 | 3.0 | 101.42 | 100.63 | 3.0 | 103.66 | 99.06 |
| 7 | 21.0 | 98.97 | 100.00 | 1.8 | 100.26 | 100.73 | 2.0 | 100.86 | 98.59 |
| 8 | 11.0 | 98.74 | 100.01 | 1.2 | 101.08 | 101.48 | 5.0 | 96.81 | 98.58 |
| 9 | 6.0 | 98.26 | 99.98 | 3.0 | 102.41 | 100.81 | 2.0 | 100.83 | 99.45 |
| 10 | 21.0 | 97.47 | 99.99 | 1.2 | 99.05 | 99.51 | 4.0 | 100.89 | 102.01 |
| 11 | 6.0 | 97.69 | 100.03 | 2.4 | 98.65 | 98.08 | 4.0 | 101.03 | 99.11 |
| 12 | 16.0 | 98.70 | 99.99 | 2.4 | 100.00 | 99.42 | 3.0 | 100.5 | 98.55 |
| 13 | 16.0 | 101.38 | 100.02 | 1.8 | 97.94 | 102.49 | 5.0 | 100.14 | 99.28 |
| 14 | 11.0 | 100.32 | 99.97 | 3.0 | 100.32 | 99.61 | 4.0 | 98.78 | 100.56 |
| 15 | 21.0 | 102.03 | 100.00 | 2.4 | 99.57 | 97.45 | 5.0 | 99.42 | 101.58 |
| **Mean ±**  **SD** | | 100.10 ±  2.030 | 100.0 ± 0.095 | **Mean±**  **SD** | 99.50 ± 1.600 | 100.10 **±** 1.650 | **Mean ±**  **SD** | 100.33 ± 1.730 | 99.62 ± 1.443 |

**Table** **S3.** Statistical comparison for the results obtained by the suggested methods and the reported method for the analysis of CIP and FLU.

| **Parameter** | **CIP** | | | | | | | **FLU** | | | |
| --- | --- | --- | --- | --- | --- | --- | --- | --- | --- | --- | --- |
|  | **D^1^** | **RD** | **DR** | **MC** | **PLS** | **ANN** | **Official method ^a^** | **DDRD** | **PLS** | **ANN** | **Official method [2]^a^** |
| **Mean of recoveries** | 100.19 | 100.89 | 100.00 | 100.28 | 99.41 | 99.78 | 100.12 | 99.53 | 100.29 | 99.98 | 100.20 |
| **S.D.** | 0.344 | 0.450 | 1.633 | 0.715 | 0.931 | 1.004 | 0.758 | 1.286 | 0.854 | 0.882 | 0.917 |
| **Variance** | 0.118 | 0.203 | 2.668 | 0.511 | 0.867 | 1.009 | 0.574 | 1.655 | 0.729 | 0.779 | 0.841 |
| **N** | 5 | 5 | 5 | 5 | 5 | 5 | 5 | 5 | 5 | 5 | 5 |
| **Degree of freedom** | 4 | 4 | 4 | 4 | 4 | 4 | 4 | 4 | 4 | 4 | 4 |
| **Student's t-test (2.306) ^b^** | 0.204 | 1.969 | 1.103 | 0.567 | 1.365 | 0.691 | ---------- | 0.948 | 0.333 | 0.246 | ------- |
| **F-test (6.388) ^b^** | 4.864 | 2.828 | 4.648 | 1.123 | 1.510 | 1.758 |  | 1.968 | 1.271 | 1.357 |  |

^a^ Official method: For determination of CIP, HPLC method was developed using octadecylsilyl silica gel column as a stationary phase with mobile phase of acetonitrile- buffer solution (13.0: 87.0, V/V) and flow rate 1.5 mL/ min and UV- detection was obtained at 278.0 nm. For determination of FLU, HPLC method was developed using octadecylsilyl silica gel column with mobile phase mixture of A= (acetonitrile- buffer solution) B= (water- acetonitrile), flow rate 1.0 mL/ min and UV- detection was obtained at 238.0 nm.

^b^ The corresponding theoretical t and F values (*p* = 0.05).

**References**

[1] Rorabacher DB. Statistical Treatment for Rejection of Deviant Values: Critical Values of Dixon’s “Q” Parameter and Related Subrange Ratios at the 95% Confidence Level. Analytical Chemistry. 1991;63:139–146.

[2] British pharmacopoeia, The Stationery Office, volume I, London, 2016.
